# Heavily glycosylated, highly fit SIVMne variants continue to diversify and undergo selection after transmission to a new host and they elicit early antibody dependent cellular responses but delayed neutralizing antibody responses

**DOI:** 10.1186/1743-422X-5-90

**Published:** 2008-08-04

**Authors:** Dawnnica Eastman, Anne Piantadosi, Xueling Wu, Donald N Forthal, Gary Landucci, Jason T Kimata, Julie Overbaugh

**Affiliations:** 1 Division of Human Biology, Fred Hutchinson Cancer Research Center, Seattle, WA, USA; 2 Program in Molecular and Cellular Biology University of Washington, Seattle, WA, USA; 3 Department of Pathobiology, University of Washington, Seattle, WA, USA; 4 Division of Infectious Diseases, University of California, Irvine, CA, USA; 5 Molecular Virology and Microbiology, Baylor College of Medicine, Houston, TX, USA; 6 Vaccine Research Center, NIAID, NIH, Bethesda, MD, USA

## Abstract

**Background:**

Lentiviruses such as human and simian immunodeficiency viruses (HIV and SIV) undergo continual evolution in the host. Previous studies showed that the late-stage variants of SIV that evolve in one host replicate to significantly higher levels when transmitted to a new host. However, it is unknown whether HIVs or SIVs that have higher replication fitness are more genetically stable upon transmission to a new host. To begin to address this, we analyzed the *envelope *sequence variation of viruses that evolved in animals infected with variants of SIVMne that had been cloned from an index animal at different stages of infection.

**Results:**

We found that there was more evolution of *envelope *sequences from animals infected with the late-stage, highly replicating variants than in animals infected with the early-stage, lower replicating variant, despite the fact that the late virus had already diversified considerably from the early virus in the first host, prior to transmission. Many of the changes led to the addition or shift in potential-glycosylation sites-, and surprisingly, these changes emerged in some cases prior to the detection of neutralizing antibody responses, suggesting that other selection mechanisms may be important in driving virus evolution. Interestingly, these changes occurred after the development of antibody whose anti-viral function is dependent on Fc-Fcγ receptor interactions.

**Conclusion:**

SIV variants that had achieved high replication fitness and escape from neutralizing antibodies in one host continued to evolve upon transmission to a new host. Selection for viral variants with glycosylation and other envelope changes may have been driven by both neutralizing and Fcγ receptor-mediated antibody activities.

## Background

Lentiviruses such as human and simian immunodeficiency viruses (HIV and SIV, respectively) are notorious for their extensive genetic variation, and for their rapid diversification within a single host [1]. In part, this diversification is due to the virus' rapid rate of replication and the high error rate of reverse transcription. However, there is also evidence that viruses evolve under selection pressure to both evade the host immune response and to achieve higher levels of replication fitness. Variants that emerge at later stages of infection tend to be more pathogenic than those found earlier, and there is some indication that virus diversification may reach a plateau late in infection [2]. It is unclear to what extent genetic variation of lentiviruses such as SIV and HIV is influenced by the properties of the infecting strain, the level of replication, or the immune response to the virus. It is also not known whether viruses that have achieved high fitness in one host continue to diversify following transmission to a new host.

The SIV/macaque model is an appealing system for examining lentiviral evolution over the course of infection because the sequence of the infecting virus and the time of infection are defined [3-5]. In previous studies, we investigated virus evolution in pig-tailed macaques infected with a cloned virus, SIVMneCL8 [6-8]. These analyses were focused on the *envelope *gene because it encodes the surface unit (SU) glycoprotein, which plays a key role in viral entry and is a target of both humoral and cellular responses [9]. These early SIV studies showed that variation occurred primarily in previously defined hypervariable domains of *envelope*, especially the first variable region (V1). In particular, there was a notable accumulation of potential N- and O-linked glycosylation sites [8]. Biochemical studies showed that these amino acid changes were, in fact, targets for the addition of carbohydrates and that such glycosylation changes allowed the virus to escape the neutralizing antibodies directed against the parental, infecting cloned virus, SIVMneCL8 [6,7]. Similar changes in glycosylation sites in SU over the course of infection have since been noted in both in the SHIV/macaque model [10-12] and in HIV-1 infection in humans [13,14].

To examine properties of viruses that emerge later in infection, prototype variants of SIVMneCL8 that evolved in infected animals at intermediate and late stages of infection were isolated and characterized [6,7,15,16]. SIVMneCL8 itself has characteristics that are similar to variants found early in HIV-1 infection of humans – it is macrophage-tropic, neutralization sensitive, and causes an infection with viral replication levels typical of HIV-1 infection in humans [7,16]. The prototype intermediate-stage virus, SIVMne35wkSU, differs from SIVMneCL8 at only four amino acid positions, all in V1, each of which are sites for carbohydrate modifications [6,7]. This intermediate-stage virus has escaped neutralizing antibodies directed against the infecting virus, SIVMneCL8 [7]. Molecular clones representing later viruses from both blood (SIVMne170) and lymph node (SIVMne027) are also antigenically distinct from the 'early' virus, SIVMneCL8, and they are more cytopathic in both primary T-lymphocytes and T-cell lines [15,16].

Animals infected with intermediate- and late-stage variants had approximately100-fold and 3,000-fold higher plasma RNA levels at set-point, respectively, than animals infected with the parental, early virus [17]. Moreover, animals that were infected with the intermediate and late-stage variants did not show evidence of having elicited neutralizing antibodies to the autologous virus at 24 weeks post-infection [17]. However, it is unknown whether antibodies that can neutralize the intermediate- and late-stage variants developed at later times in infection, perhaps at levels that would not have been detected in previous studies where a relatively stringent 90% cut off was applied to define neutralization. Nor was there any information on non-neutralizing antibody activities, such as those mediated by Fc-Fcγ receptor (FcγR) interactions, which is detected early and that correlates with the decline in viremia during acute HIV infection [18].

Given that the intermediate and late-stage variants replicated to high levels in these animals in the apparent absence of a neutralizing antibody response, we wondered whether the viruses continued to evolve in a manner similar to that observed for SIVMneCL8. To begin to define how the fitness of the infecting virus strains affects subsequent viral diversification in the host, we analyzed sequence variation of the V1–V3 region of *envelope *over time in the animals infected with early-, intermediate- and late-stage viruses. We also assessed the neutralizing antibody response at later times in infection and examined associations between antibody responses, viral load, and virus diversification. In addition, we examined antibody-dependent virus inhibition (ADCVI) activity, an FcγR-dependent antibody response, that we hypothesized could play a role at earlier stages of infection based on findings in HIV-1-infected humans [18].

## Results

### Intermediate and late-stage SIV variants continue to diverge upon transmission to a new host

In a previous study, SIV variants were isolated from an infected animal at early, intermediate, and late stages of infection, and these sequential variants were used to infect a new set of macaques [17]. To evaluate the evolution of SIV variants upon transmission to a new host, we compared *envelope *(*env*) V1–V3 sequences from each of two animals infected with the early (SIVMneCL8), intermediate (SIVMne35wkSU) and late-stage (SIVMne170 and SIVMne027) variants. We cloned V1–V3 *env *sequences from PBMC DNA from two times post-infection, 40 weeks and approximately 75 weeks (71–77 weeks depending on the sample available), both of which were after the immune response to the virus had a chance to develop, but before most of the animals had overt AIDS. To avoid resampling bias, we obtained a total of 8–12 clones from 2–3 independent low-copy PCRs from each sample. We examined a median of 16 sequences per animal per time point (range 10–42).

**Figure 1:**
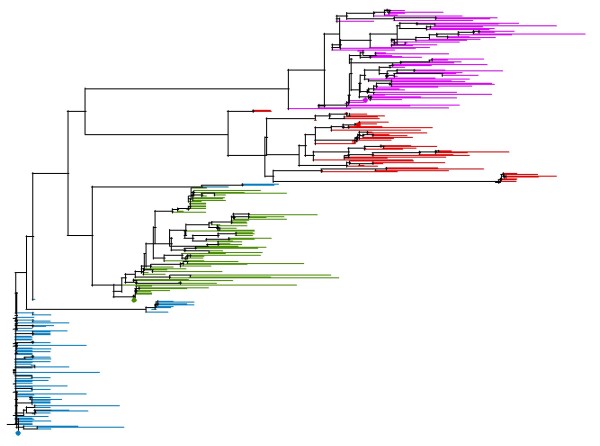
Phylogenetic relationship of viral variants. A distance-based tree was created using all unique sequences from all animals at both 40 and approximately 75 weeks-post infection. Sequences from SIVMneCL8-infected animals are shown in blue, those from SIVMne35wkSU-infected animals are shown in green, and those from SIVMne170- and SIVMne027-infected animals are shown in red and pink respectively. The parental sequences are marked by a diamond of the respective color.

A phylogenetic tree was generated from all unique sequences from all animals (Figure 1). In general, sequences from animals that were infected with the same initial variant clustered together. Within these clusters, sequences from the same animal grouped together (not labeled). Sequences from animals infected with SIVMneCL8 tended to be less divergent than sequences from animals infected with SIVMne35wkSU, which were less divergent than sequences from animals infected with SIVMne170 and SIVMne027. As an exception, some sequences from an SIVMneCL8-infected animal grouped with the SIVMne35wkSU sequences; while we cannot rule out contamination, this could also be the result of convergent evolution.

**Figure 2:**
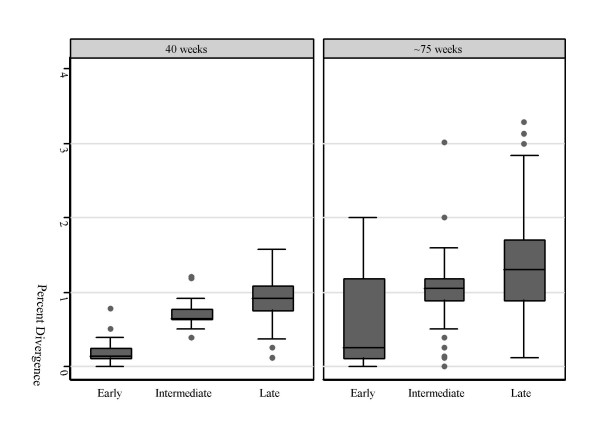
Percent divergence from the infecting variant. For each animal, genetic distances were calculated between each sequence and the infecting variant, and animals were grouped by infecting variant. Box plots show the divergence of sequences from animals infected with the early variant (SIVMneCL8), the intermediate variant (SIVMne35wkSU), and the late variants (SIVMne170 and SIVMne027), at 40 and ~75 weeks post-infection.

For each animal, we calculated the average diversity and the average divergence from the infecting clone for sequences sampled at both 40 and ~75 weeks post-infection. For purposes of comparison, we grouped the results of the animals infected with the two different late-stage variants together, as animals infected with both late-stage viruses had similar viral loads (Table 1) and disease course [17]. As shown in Table 1, at week 40 the average SIV sequence diversity in animals infected with the early variant, SIVMneCL8, (0.39%) was lower than that in animals infected with either the intermediate variant (1.20%) or the late variants (1.05%). At ~75 weeks after infection, a similar trend was observed, and diversity has increased in all groups. At this time, the average diversity was 0.96% in animals infected with the early variant, 1.39% in animals infected with the intermediate variant, and 1.69% in animals infected with the late variants. We also calculated the divergence of each SIV sequence from the infecting variant at both 40 and ~75 weeks post-infection, as shown in Figure 2. At 40 weeks, the average divergence was 0.20% for animals infected with the early variant, 0.71% for animals infected with the intermediate variant, and 0.94% for animals infected with the late variants. At ~75 weeks, the average divergences had increased to 0.60%, 1.17%, and 1.44%, respectively. We assessed whether the extent of virus evolution was significantly higher in animals infected with the late variants (SIVMne170 and SIVMne027) compared to animals infected with SIVMneCL8 using a Mann-Whitney U test. At 40 weeks post-infection, there was a trend for increased diversity in animals infected with the late variants (p = 0.07), while at ~75 weeks post-infection, this relationship was not significant (p = 0.17). At both 40 and ~75 weeks post-infection, there was a trend for increased divergence in animals infected with the late variants (p = 0.06 and p = 0.07, respectively).

**Table 1:** Virus evolution in animals infected with different variants.

					**Week 40**			**Week ~75**		
						
**Infecting variant**		**Animal**	**Set point viral load^1 ^** **(log10 copies/mL)**	**NtAb^2 ^peak IC50**	**Diversity^3^**	**Mean Divergence^4 ^(Range)**	**Mean dN/dS**	**Diversity^3^**	**Mean Divergence**^4 ^**(Range)**	**Mean dN/dS**
"Early"	SIVMneCL8	J95155	3.2	759	0.40	0.2 (0–0.77)	0.16	0.52	0.52 (0–2.00)	1.07
		F95274	3.8	1133	0.38	0.2 (0–0.51)	0.17	1.39	0.69 (0–1.35)	0.54
		**Average**	**3.5**	**946**	**0.39**	**0.20**	**0.16**	**0.96**	**0.60**	**0.81**

"Intermediate"	SIVMne35wkSU	J95251	5.1	20				1.05	0.85 (0–1.48)	1.02
		J96165	6.1	53	1.20	0.71 (0.38–1.21)	0.96	1.73	1.49 (0.90–3.01)	1.22
		**Average**	**5.6**	**36.5**	**1.20**	**0.71**	**0.91**	**1.39**	**1.17**	**1.12**

"Late"	SIVMne170	F94393	6.9	1229	0.68	0.79 (0.51–1.17)	1.09	1.51	1.26 (0.13–2.00)	1.33
		J94233	6.5	266	1.22	1.28 (0.89–1.57)	1.93	2.32	2.18 (0.38–3.28)	1.57
	SIVMne027	J94454	7.0	1278	1.03	0.7 (0.13–1.16)	0.67	1.32	0.91 (0.51–1.43)	1.00
		K94379	6.9	1517	1.27	1 (0.77–1.29)	1.16	1.59	1.4 (0.51–2.40)	1.07
		**Average**	**6.8**	**1072.5**	**1.05**	**0.94**	**1.21**	**1.69**	**1.44**	**1.24**

^1 ^Set point viral load[17]

^2 ^NtAb peak IC50 = highest reciprocal dilution of plasma needed to neutralize 50% of virus infectivity

^3 ^NtAb Diversity = average pairwise distance

^4 ^Divergence = distance from infecting strain.

**Figure 3:**
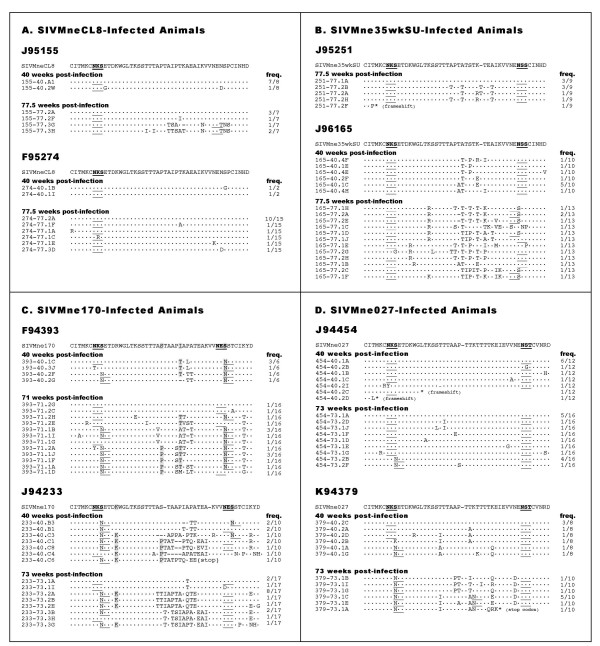
V1 sequence variants. Amino acid sequence data from the V1 region of envelope is shown for each animal at each time point analyzed. Each sequence represents a unique variant and the frequency with which it was observed is shown in the column to the right. The parental V1 sequence is shown at the top of each alignment, and the conserved amino acids in each variant sequence are shown as dots. Sites of potential N-linked glycosylation are underlined in each sequence, and positions of reversion to the amino acid found in SIVMneCL8 are highlighted in grey.

We were interested in determining whether the nucleotide changes that arose in animals infected with the intermediate and late variants reflected continued diversification or reversion towards a more ancestral state [19]. We calculated the average divergence from the SIVMneCL8 sequence for sequences from each animal at both 40 and ~75 weeks post-infection. The infecting clone SIVMne35wkSU is 0.8% divergent from SIVMneCL8, and animals infected with this variant had an average divergence from SIVMneCL8 of 1.41% at week 40 and 1.76% at week ~75. The infecting clones SIVMne170 and SIVMne027 are 2.56% and 2.70% divergent from SIVMneCL8, respectively; animals infected with these clones had average divergences of 2.65% and 3.18% from SIVMneCL8 at week 40 and 3.44% and 3.45% at week ~75. Thus, we did not observe any general reversion towards the ancestral SIVMneCL8 sequence. As shown in Figure 3, we observed several specific amino acid positions in the majority of sequences from animals infected with SIVMne170 that reverted to the amino acid present in SIVMneCL8. For example, position 120 was a K in SIVMneCL8 and an R in SIVMne170, and reverted to a K in animal J94233 (19/27 sequences). Position 137 was a T in SIVMneCL8 and an I in SIVMne170, and reverted to a T in most sequences from animal F94393 (13/22 sequences), and was highly variable in J94233. Overall, however, virus populations continued to diverge, and the average divergence from SIVMneCL8 was in fact greater in animals infected with late variants compared to animals infected with SIVMneCL8, although there was only a trend for statistical significance (Mann-WhitneyU test, p = 0.06 for week 40, p = 0.07 for week ~75).

### Intermediate and late variants have higher nonsynonymous divergence

Because the intermediate and late-stage variants replicate to higher levels than SIVMneCL8, they could achieve a higher level of diversity and divergence due to the random accumulation of changes throughout many rounds of virus replication. To determine whether the increased virus evolution observed among animals infected with the intermediate and late variants was due to random accumulation of changes or selection, we calculated the ratio of nonsynonymous to synonymous changes (dN/dS) between each sequence and the infecting variant using SNAP [20]. As shown in Table 1, the average dN/dS ratio was <1 for animals infected with SIVMneCL8 at both 40 and ~75 weeks post-infection (0.16 and 0.81 respectively), indicating purifying selection. By contrast, the dN/dS ratio was close to or higher than 1 for animals infected with the intermediate and late variants, indicating a lack of selection or in some cases evidence for positive selection. We evaluated whether the average dN/dS ratio was significantly higher among animals infected with the late variants (SIVMne170 and SIVMne027) compared to animals infected with SIVMneCL8. We found a trend for significance at 40 weeks (Mann-Whitney p = 0.06) and no association at ~75 weeks (Mann-Whitney p = 0.24).

We also separately evaluated the nonsynonymous and synonymous divergence of each sequence compared to the infecting variant. For each animal, we calculated the average nonsynonymous divergence and synonymous divergence in the *envelope *sequences. At 40 weeks post infection, *envelope *sequences from animals infected with the late variants had marginally significantly greater nonsynonymous divergence than animals infected with the early variant (Mann-Whitney p = 0.06), however there was no difference in synonymous divergence (Mann-Whitney p = 0.16). At ~75 weeks post infection, the difference in both nonsynonymous and synonymous divergence was marginally significant (Mann-Whitney p = 0.06). Together, these results indicate that difference in virus evolution between animals infected with the late-stage variants versus early-stage variants can not be explained entirely by a higher level of error-prone virus replication.

### Intermediate and late variants evolve more changes in length and glycosylation sites

We next compared specific sequence features that may be associated with adaptive evolution: length variation and changes in potential glycosylation sites. We calculated the length of the *env *V1–V3 region for each sequence and determined the number of sequences from each animal that differed in length from the infecting variant (data not shown). For animals infected with the early variant, there was no length variation at 40 weeks and little length variation at ~75 weeks (7% in one animal). Some animals infected with the intermediate and late variants also had no length variation, while others demonstrated extensive variation (up to 80% in one animal). Although animals infected with the late variants had more length variation than animals infected with the early variant, this difference was not statistically significant (Mann-Whitney p = 0.14).

We calculated the percent of potential N-linked glycosylation sites (PNGS) that were added, deleted or shifted to an adjacent residue. In the animals infected with the early and intermediate-stage variants, no PNGS changes were detected at 40 weeks post-infection; in the late-stage virus-infected, 0.7% to 9.4% varied. We saw the same pattern at ~75 weeks after infection, when 0.4% to 2.2% of the PNGS varied in sequences from animals infected with the early-stage virus and 2.2% to 15.1% varied in the sequences from the animals infected with late-stage variants.

Because most of the amino acid differences that we observed were in the V1 region, we performed a more detailed analysis of this region. We compared the amino acid sequences of all the unique variants detected at 40 and ~75 weeks PI to the sequence of the infecting variant (Figure 3). Extensive variation in V1 was evident as early as 40 weeks PI in some animals, including changes that would be predicted to create sites of N and O-linked glycosylation. In general, we observed very little variation in the animals infected with SIVMneCL8 compared to animals infected with the intermediate and late variants.

There were several common changes that were present in viruses from animals infected with the late-stage variants. There was a shift in a potential N-linked glycosylation site (PNGS; a sequence of NxT/S) from position 114 to 116 (positions are based on the sequence of SIVMneCL8 envelope surface unit) in viruses from all the animals infected with SIVMne027 and SIVMne170. However, this shift in PNGS from 114 to 116 was not detected in any of the variants from animals infected with either early or intermediate-stage virus. In animals infected with SIVMne170, there was an additional shift in PNGS from position 146 to 148.

There was also considerable variation in the mid region of V1, between the two variable PNGS noted above, particularly in animals infected with SIVMne170. This is a region of V1 in which we previously identified sites of O-linked glycoslyation [6]. In virus from animals infected with SIVMne170, there were a number of changes to threonine and to a lesser extent, to serine, further enriching this region within the center of V1 with potential targets for O-linked carbohydrates. Interestingly, relatively few changes in SIVMne027 were to serine and threonine, perhaps reflecting the fact that the infecting parental virus, SIVMne027 had the highest number of serine and threonines in this region (N = 11) relative to the other infecting viruses (N = 7–9). In contrast, there were more serine and threonine changes in viruses from animals infected with SIVMne35wkSU, a virus with lower density of these amino acids in the infecting virus (N = 9). Surprisingly, there were relatively few changes in V1 in animals infected with SIMneCL8, which had the fewest initial serine/threonine (N = 7), except in three clones from one animal (J95155) at 77 weeks. These changes at 77 weeks, which included 4 additional serine/threonine (in addition to a threonine to create the PNGS at 146), created a cluster of residues reminiscent of those that evolved to create the SIVMne35wkSU variant in another animal infected with SIVMneCL8 [5].

### Neutralizing antibody (NtAb) responses develop later in animals infected with late-stage variants

The extensive variation within V1 in animals infected with the intermediate and late-stage variants was unexpected because we could not detect any SIV-specific neutralizing antibodies against the infecting virus at 24 weeks post-infection in any of the macaques infected with these variants (<90% neutralization at 1:4). In contrast, both macaques infected with the early-stage virus, SIVMneCL8, generated neutralizing antibodies at levels typical of SIVMne infections (>90% neutralization at 1:64) during that same period [17].

**Figure 4:**
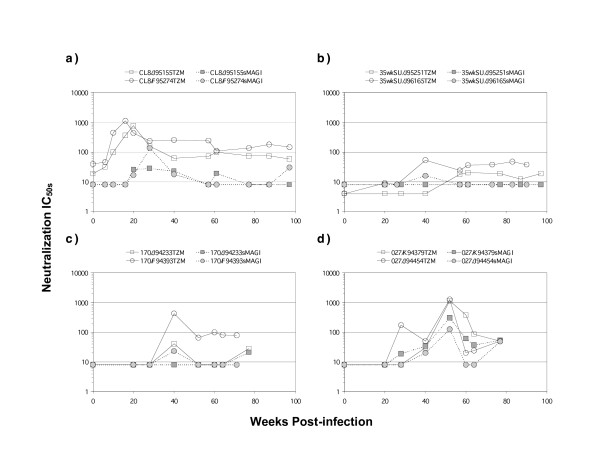
Neutralization IC_50_s of sera collected at various times after infection against the infecting virus. Each panel represents neutralization of macaques infected with the same virus: a) SIVMneCL8; b) SIVMne35wkSU; c) SIVMne170; d) SIVMne027. For each panel, the x axis shows the time when sera samples were collected. The y-axis shows the IC_50 _– the reciprocal dilution of sera required to inhibit infection by 50%. Neutralization IC_50_s measured by the TZM-bl cells are shown in solid lines. Neutralization IC_50_s measured in sMAGI cells are shown in dotted lines.

To understand the kinetics of NtAb responses in each of the infected monkeys, we assessed sera taken from various times after infection for neutralization activity against the inoculating virus, using a cell line TZM-bl [21] that expresses CD4 and CCR5 and that is highly susceptible to infection by all the SIVMne variants (not shown). For comparison, we also examined neutralization using the sMAGI indicator cells [6], which express CD4 and endogenous coreceptor, because we had used these cells for neutralization assays in our previous study [17]. The neutralization activity was measured as the 50% inhibition concentration (IC_50_), which is the reciprocal of sera dilution that is required to inhibit viral infection by 50%. As shown in Figure 4a, both monkeys infected with SIVMneCL8 developed comparable NtAb kinetics, which peaked as early as 16–20 weeks post-infection, with potent IC_50 _titers around 1,000 using the TZM-bl cell assay. After the initial peak, the NtAb IC_50 _titers against the infecting virus were maintained at ~100. The SIVMne35wkSU-infected monkeys had much lower IC_50 _NtAb titers (Figure 4b) than SIVMneCL8-infected monkeys, and there was no NtAb detected before 40 weeks post-infection. Similarly, neither SIVMne170-infected monkey had detectable NtAbs before 40 weeks post-infection. However, the NtAb titer was relatively high (IC_50 _of 431) at 40 weeks post-infection in monkey F94393, and subsequently declined (IC_50_s of 65 – 99) after 40 weeks post-infection. The second SIVMne170-infected animal, J94233, had a low but detectable NtAb responses at 40 and 77.5 weeks PI (Figure 4c). The NtAb responses in both SIVMne027-infected monkeys were comparable and peaked at 52 weeks post-infection (IC_50_s of 1,161 and 1,278; Figure 4d).

The average NtAb IC_50 _was not significantly higher for animals infected with the late variants (SIVMne170 and SIVMne027) compared to animals infected with SIVMneCL8 at either 40 weeks (Mann-Whitney p = 1.00) or ~75 weeks (Mann-Whitney p = 0.35). The average peak IC_50 _was also not significantly different (Mann-Whitney p = 0.35), however the peak IC_50 _occurred later among animals infected with the late variants (Mann-Whitney p = 0.06). The contemporaneous NtAb IC_50 _was not associated with virus evolution at either 40 weeks or ~75 weeks post-infection (Spearman's rho = -0.43, p = 0.34 for diversity vs IC_50 _at 40 weeks; Spearman's rho = 0.05, p = 0.91 for diversity vs IC_50 _at ~75 weeks; Spearman's rho = 0.09, p = 0.85 for divergence vs IC_50 _at 40 weeks; Spearman's rho = -0.02, p = 0.96 for divergence vs IC_50 _at 40 weeks). The peak NtAb IC50 was also not associated with viral load set point (Spearman's rho = 0.61, p = 0.15).

Because we did not detect NtAb through 24 weeks PI in some animals in our previous study using the sMAGI cells as targets for infection [17], but we did detect responses at later times using the TZM-bl cells here, we also examined neutralization by sera at different times post-infection using sMAGI cells. In general, the IC_50 _titers determined using sMAGI cells were lower than those determined using TZM-bl cells. For example, the neutralization titers were at least several-fold, and in some cases more than 10-fold lower in the sera of SIVMneCL8-infected animals at almost every time point tested. There were also notable differences in the IC_50 _values using these two cell lines for the animal 94393 infected with SIVMne170 (Figure 4c), but not for either animal infected with SIVMne027 (Figure 4d). The IC_50 _values were also consistently higher for animals infected with SIVMneCL8 throughout the course of infection, particularly at the earliest time points, using the TZM-bl cells as targets for infection. In contrast, NtAb responses were low to absent up through 24 weeks PI in animals infected with the intermediate- and late-stage variants using both sMAGI and TZM-bl cells.

### ADCVI antibody activity is detected in all animals

**Figure 5:**
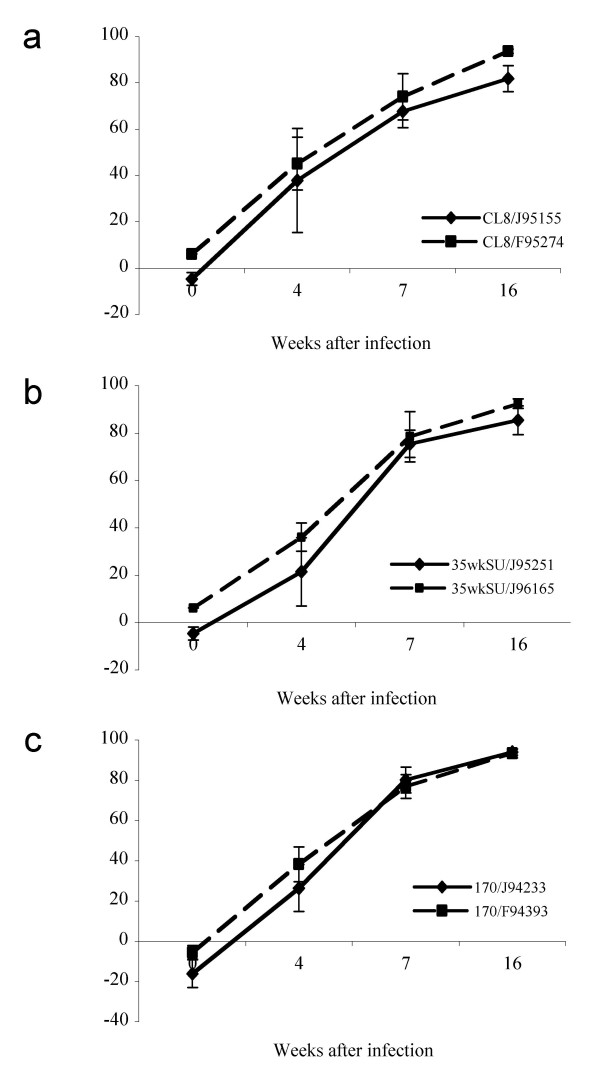
ADCVI antibody activity in plasma of infected animals. Each panel represents th ADCVI activity (% inhibition) in 1:100 dilutions of plasma from animals infected with the same virus: (a) SIVMneCL8; b) SIVMne35wkSU; c) SIVMne170. Data are means ± standard error of four measurements for each animal at each timepoint.

Previous studies have shown that ADCVI antibodies, which inhibit virus in the presence of Fcγ receptor-bearing effector cells, can be detected very early in acute HIV infection[18]. To determine whether ADCVI antibody was present prior to the evolution of potential sites for N- and O-linked glycosylation in animals infected with the different variants, we examined these antibodies in plasma samples taken at 4, 7 and 16 weeks PI infection in animals infected with SIVMneCL8, SIVMne35wkSU and SIVMne170 (Figure 5). In all animals, greater than 50% inhibition by plasma at a dilution of 1:100 could be detected by 7 weeks PI, and greater than 80% was detected by 16 weeks PI. There was very little difference in the timing or magnitude of this activity in animals infected with the different variants, suggesting that all three infecting viruses were capable of eliciting ADCVI antibodies.

## Discussion

This study is the first to directly compare the evolution of primate lentiviral variants from different stages of infection after they were transmitted to a new host. This analysis focused on envelope diversification in animals infected with molecularly cloned SIVs from early, intermediate and late stages of infection in the index animal. Surprisingly, the highest amount of diversity and divergence from the parental strain was observed in animals infected with the late-stage viruses, despite the fact that this infecting virus had already been selected for fitness and persistence in the first animal. In all measures of sequence evolution we analyzed and at each time point, virus evolution was highest in animals infected with the late-stage virus compared to the early-stage virus. The primary limitation of this study was the small number of animals included (2 animals were infected with the early-stage variant, two with the intermediate-stage variant, and four with the late-stage variants). However, despite the small sample size, many of the differences that we observed in virus evolution and neutralizing antibody response approached statistical significance. Envelope variation, including changes in glycosylation, was detected in some cases prior to the detection of neutralizing antibodies, but after the development of an ADCVI antibody response.

The late variants replicate to higher levels than the early virus and thus undergo more rounds of error-prone reverse transcription. However, this increased replication is not likely to completely explain the higher envelope diversification of the late-stage viruses because the dN/dS ratio was higher among animals infected with intermediate and late-stage viruses compared to animals infected with the early virus, at least at 40 weeks post-infection. In addition, there was greater nonsynonymous divergence but not synonymous divergence in animals infected with the intermediate and late variants. Finally, animals infected with intermediate and late variants had more length changes and more changes in potential glycosylation sites.

We observed that the virus populations in these animals continued to evolve away from the ancestral SIVMneCL8 strain, rather than reverting to an ancestral state. Reversion has been observed in other studies of SIV [22,23] and HIV [19,24]. However, in these studies, reversion was generally observed at earlier times in infection than was examined in this study. Furthermore, ours was the only study to compare virus evolution with respect to a known, cloned ancestor, SIVMneCL8, allowing us to examine reversion to known ancestral sequences, rather than predicted sequences.

There were some common changes among viruses from different animals, particularly those infected with the late-stage variants. These included shifts in PNGS at both the N (114–116) and C terminus (146–148) of the V1 domain. While the shift at the N terminus was found in all four animals infected with the late-stage variants, such a shift was not detected in any of the variants from animals infected with either early or intermediate-stage virus, suggesting the advantage of this PNGS may be context dependent. There was no C-terminal PNGS in the original parental SIVMneCL8, but a PNGS was present in the intermediate and late-stage variants. Previous biochemical analyses have verified that the predicted glycosylation site in the C-terminus of V1 is indeed a target of carbohydrate addition [6]. The changes between these two PNGS included several that would be predicted to add sites for O-linked carbohydrates; in all animals, variants evolved that had almost half of the amino acids in this mid region of V1 as serine and threonine. In general there tended to be more changes of this type in viruses from animals infected with a variants that had fewer serines or threonines initially.

In previous studies, we showed that at 24 weeks PI, NtAb were detectable in SIVMneCL8-infected animals, but not animals infected with the intermediate or late-stage viruses. The present studies, which include additional follow-up, showed that animals infected with the late-stage variants did eventually develop NtAb, and in some cases, at titers comparable to those of SIVMneCL8-infected animals. There was some variation in the magnitude of the NtAb response depending on the assay that was used – TZM-bl or sMAGI cells. In general, the assays gave qualitatively similar results, although values with TZM-bl assay were almost always higher and more so with sera from some animals versus others. It is not clear why there were quantitative differences in these assays that appear dependent on the virus and serum combination tested, but it could reflect differences in the coreceptor used for entry in the two cell lines: CCR5 for TZM-bl cells and an unknown coreceptor for sMAGI cells [25].

In animals infected with the late-stage variants, NtAb responses were delayed, with peak responses at 40–52 weeks in animals infected with late-stage variants versus 16–20 weeks in animals infected with the early virus. In general, NtAb responses were poor in animals infected with the intermediate virus, although low levels could be detected starting at 40–60 weeks. Thus, it is possible that neutralizing antibody responses to the intermediate and late-stage viruses, which have a higher density of glycosylation sites in V1, are delayed due to the fact that key epitopes are shielded by carbohydrates. The fact that responses eventually develop suggests that this shield is not impenetrable.

There was no obvious relationship between the extent of virus variation and the timing or magnitude of NtAb response. For example, as noted, animals infected with SIVMnCL8 had the earliest peak NtAb responses, but showed little divergence and diversity at either 40 or ~75 weeks after infection. Animals infected with the intermediate and late-stage variants had later peak NtAb responses, but demonstrated considerable virus evolution before this peak, at 40 weeks after infection. The magnitude of the NtAb response was not associated with either virus evolution or viral load at 40 or ~75 weeks after infection.

The peak NtAb response was also not associated with the viral load set point. This is in contrast to findings in one study of chronically HIV-infected humans, where there was a negative correlation between NtAb responses to autologous virus and viral load [26]. It is worth noting that this association was observed when examining contemporaneous virus and antibody and from variable times in infection using a cross sectional study design. In contrast, our study specifically examined NtAb response to the infecting virus and the relationship of these NtAb responses to viral levels. In a separate study, HIV-1 neutralization was positively correlated with viral load among individuals infected with nef-attenuated viruses, while no association was observed among a control cohort [27]. Thus, the relationship between neutralizing antibody response and viral load remains unclear and may depend on other factors. Our finding of no relationship between these factors is consistent with a model proposed by Frost et al. [28], in which antibodies may exert "soft" selection pressure that promotes the survival of certain virus variants but not others, while not affecting the absolute level of virus replication. However, it is important to note that the small sample size of our study may limit our ability to identify more subtle relationships between NtAb and viral set point.

In some cases, the variation that was observed in V1, including changes in PNGS, was detected prior to the detection of NtAbs. This is best exemplified in animals infected with SIVMne170 virus. In these animals, shifts in PNGS, as well as changes in a region thought to be a target of O-linked glycosylation [6], were observed by 40 weeks PI. However, NtAb could not be detected in advance of this time, and were quite low even at 40 weeks and thereafter in one animal, 94233, who had viruses with extensive variation in V1 at 40, 52 and 73 weeks PI. This may suggest that other selective pressures are important in driving these changes. Given that the parental, infecting virus SIVMne170 was already highly fit for replication, as evidenced by viral set point levels of >10^6^, it seems unlikely that the changes are simply ones that permit high replication fitness. We also consider it somewhat unlikely that selection was driven by T cell responses because the types of changes – addition of carbohydrates – and the cluster of multiple changes is not particularly characteristic of changes in epitopes that are targeted by CD4 or CD8 T cells; however, we cannot rule out this possibility. Notably, we found that ADCVI antibody responses were present prior to the detection of this variation. Indeed, potent ADCVI was detected by 7–16 weeks post-infection in all animals examined, irrespective of the sequence of the infecting virus. Thus, it is tempting to speculate that ADCVI activity may have played a role in selecting viruses with these gylcosylation changes because these responses were observed very soon after infection and well before viruses with these changes were observed. Furthermore, ADCVI or other FcγR-mediated antibody functions have been shown to be active against lentiviruses in vitro [29]. Nonetheless ADCVI alone does not explain why there were so few V1 changes in animals infected with the early-stage virus, because these animals also developed ADCVI antibody within the first few months of their infection. Moreover, these animals had the earliest NtAb responses. This suggest a combination of the level of virus replication, the properties of the virus itself, and various B cell responses may contribute to the evolution of glycosylation changes.

## Conclusion

This study of macaques infected with highly related, cloned SIVs allowed a unique window into the relationship between infecting viral strain, viral evolution and immune responses. We found that SIV variants that had achieved high replication fitness and escape from neutralizing antibodies in one host continued to evolve upon transmission to a new host. Furthermore, we observed evidence that this evolution was not merely the result of a high replication level, but included changes that were similar between animals and that had plausible biological consequences. The timing of virus diversification suggests that neutralizing antibodies are unlikely to have played a major role in selecting these changes, while ADCVI antibodies might have been an important source of early selective pressure. Overall this study indicates that virus evolution is a continual process that is largely shaped by selective pressures that remain poorly understood.

## Methods

### Cloning of SIV envelope sequences

DNA was extracted from viably frozen macaque PBMC samples, obtained from a previous study [17], using the Qiagen Blood Mini kit and the resulting DNA was stored in aliquots at -20°C until use. All DNA purification steps were performed in a separate lab, free from any amplified DNA products to reduce the possibility of contamination from other sequences in the subsequent PCR steps.

Nested PCR was performed to amplify *env *sequence encompassing the variable regions as described previously [5], except the second round PCR product was amplified using SIVenv 37 and SIVenv 50 primers (5' GCCTTGTGTAAAATTAACCCC3' and 5' GGATGTTTGACAATGGTCTG 3' respectively). The resulting product spanned sequences encoding V1–V3 of SIVMne envelope. Five to ten proviral copies, as quantified by real-time PCR [30], were added to each first round PCR. The PCR products were cloned into the pCR2.1 TOPO TA vector (Invitrogen) according to the manufacturers protocol and sequenced using standard procedures.

### Phylogenetic analysis

Sequences from all animals were aligned using Clustal [31] and manually edited using MacClade [32], and no sequence regions were removed from analysis. Nucleotide distances were calculated using a GTR model in PAUP*4.10b [33]. Diversity was calculated as the mean distance between sequences from the same animal and time point. Divergence was calculated as the mean distance to the infecting variant. A neighbor-joining phylogenetic tree was constructed from all unique sequences using PAUP*4.10b. For each animal at each time point, the average nonsynonymous and synonymous divergence from the infecting variant were calculated using SNAP [20]. The average dN/dS ratio was calculated for each animal at each time point, using all sequences with non-zero synonymous divergence.

### Viral stocks and neutralization assay

The titer of SIV stocks used for neutralization assays was determined by infecting either TZM-bl [21] or sMAGI [6] cells by directly counting β-galactosidase-positive "blue" foci at 48 h postinfection. To perform neutralization assays, TZM-bl or sMAGI cells were seeded in 96-well tissue culture plates at 1 × 10^4 ^cells per well one day before infection. About 250 infectious particles were incubated with two-fold serial dilutions of heat-inactivated sera, or with growth medium alone, in a total volume of 50 μl at 37°C for 1 h. The virus-serum mix was then incubated with the pre-seeded TZM-bl or sMAGI cells at 37°C for 2 h in the presence of 20 μg/ml diethylaminoethyl-dextran. After the incubation, an additional 100 μl growth medium was added into each well. In 48 h, infection levels were determined using Galacto-Light Plus (Applied Biosystems, Foster City, CA), a quantitative assay measuring β-galactosidase activity present in the cell lysate. Infections were performed in triplicate within each experiment, and the results shown are averages from at least two independent experiments. Differences between β-galactosidase activity in the presence of sera and growth medium alone were calculated as the percentage of neutralization. No neutralization activity above 50% was observed with sera from before inoculation. The 50% inhibitory concentration (IC_50_) was calculated from a dose-response curve using the logarithmic function of Microsoft Excel and is expressed as the reciprocal dilution of serum required to inhibit infection by 50%. The highest concentrations of sera tested were 1:8 for monkeys infected with the SIVMne35wkSU virus, and 1:16 for other study monkeys.

### Detection of ADCVI antibody

ADCVI antibody activity was measured using methods similar to those described previously [18,29,34]. Briefly, target cells (CEM.NKR.CCR5 cells obtained from the NIH AIDS Research and Reference Reagent Program) were infected with virus (SIVMneCL8, SIVMne35wkSU or SIVMne170) for 96 hours and washed. Plasma from infected animals or from an uninfected control animal was then added to attain a final concentration of 1:100. In addition, fresh human peripheral blood mononuclear effector cells were added at an effector:target ratio of 10:1. Seven days later, p27 was measured in the supernatant fluid by ELISA. ADCVI activity is reported as percent virus inhibition: 100 [1 - ([p27t]/[p27n])], where [p27t] and [p27n] are the concentrations of p27 in supernatant fluid from wells containing test plasma or negative control plasma, respectively. Each plasma sample was assayed in triplicate on two separate occasions using a total of four different effector cell donors.

### Statistics

Average nucleotide diversity and divergence, dN/dS, and NtAb IC50 were compared between animals infected with the late variants versus animals infected with the early variant using a Mann-Whitney U test. Correlations between virus evolution, viral load and NtAb IC50 were performed using Spearman's correlation test. Statistical analyses were conducted using STATA 9 [35].

## Competing interests

The authors declare that they have no competing interests.

## Authors' contributions

DE developed the methods and obtained the *env *sequences, performed analyses, and provided input on the manuscript. AP performed data analysis and helped write the manuscript. XW performed the neutralization assays. DNF helped design and oversee that ADCVI assays and provided comments on the manuscript. GL performed ADCVI assays. JTK helped direct the initial infection studies and provided input and comments on the manuscript. JO contributed to the design of the study, oversaw the experiments, and wrote the manuscript.
